# Primary Mediastinal Germ Cell Tumor in a Woman Presenting With Superior Vena Cava Syndrome: A Case Report

**DOI:** 10.7759/cureus.91399

**Published:** 2025-09-01

**Authors:** Alex Abouafech, Nicholas Lorenz, John Stauffer, Oscar Milian Cardoso

**Affiliations:** 1 School of Osteopathic Medicine, Lake Erie College of Osteopathic Medicine, Bradenton, USA; 2 Department of Internal Medicine, Baptist Health, Jacksonville, USA

**Keywords:** primary mediastinal germ cell tumor, superior vena cava (svc) syndrome, svc compression, thrombosis of the internal jugular vein, unexpected malignancy

## Abstract

Mediastinal germ cell tumors (MGCTs) are rare neoplasms that typically occur in young men, with nonseminomatous subtypes predominating in this population. The occurrence of such tumors in women is exceedingly uncommon, accounting for a very small fraction of all extragonadal germ cell tumors (GCTs). We present a rare case of a 44-year-old woman diagnosed with a primary mediastinal germ cell tumor (PMGCT) complicated by superior vena cava (SVC) syndrome. The patient initially presented with severe respiratory distress, chest pain, and signs of venous congestion. Imaging revealed a large anterior mediastinal mass compressing the SVC. She was started on systemic chemotherapy with cisplatin and bleomycin, with partial symptomatic relief. Her hospital course was further complicated by anemia, volume overload, and pulmonary infiltrates. This case underscores the importance of considering MGCTs in the differential diagnosis of anterior mediastinal masses in women and highlights the potential for life-threatening complications such as SVC syndrome. Prompt recognition and multidisciplinary management are essential to improving outcomes in these rare but aggressive tumors.

## Introduction

Primary mediastinal germ cell tumors (PMGCTs) are rare neoplasms that account for only 1%-3% of all germ cell tumors (GCTs) and 15% of all mediastinal tumors [1]. They typically arise in young men and are thought to originate from aberrantly migrated germ cells during embryogenesis. Primary mediastinal germ cell tumors (PMGCTs) are rare malignancies constituting approximately 3%-15% of the mediastinal tumors. Histologic and serologic properties of PMGCTs are similar to those of gonadal germ cell tumors (GCTs) yet with poorer prognosis. These tumors occur more frequently in men with a ratio of 9/1 [2].

PMGCTs can grow rapidly and exert mass effects on mediastinal structures, leading to serious complications such as superior vena cava (SVC) syndrome, internal jugular vein thrombosis (IJVT), and pulmonary compromise. IJVT can result from the direct compression of certain malignancies (i.e., Trousseau’s syndrome), in which procoagulant factors such as tissue factor activate the coagulation cascade and lead to hypercoagulability [3]. SVC syndrome results from the obstruction or compression of the superior vena cava, which impairs venous return from the head, neck, and upper extremities. Clinical features include facial and upper extremity swelling, dyspnea, cough, orthopnea, and prominent neck or chest wall veins [4]. In severe cases, cerebral edema and hemodynamic instability may occur.

Nonseminomatous PMGCTs, which comprise the majority of these tumors in the mediastinum, are particularly aggressive and are associated with a poorer prognosis compared to their gonadal counterparts [5]. Despite treatment with platinum-based chemotherapy, outcomes remain suboptimal, especially when diagnosis is delayed or when significant cardiopulmonary compromise has already developed. Given their rarity in women and potential for rapid deterioration, PMGCTs should be considered in the differential diagnosis of anterior mediastinal masses, particularly in the setting of SVC syndrome or unexplained venous thromboses.

## Case presentation

A 44-year-old woman with no significant past medical history was diagnosed with a nonseminomatous primary mediastinal germ cell tumor (MGCTs), with metastases to lymph nodes, the bone, and possibly the liver. She initially presented with a two-week history of severe respiratory distress, chest pain, fever, and headaches. Prior to presentation, a computed tomography (CT) scan performed for the evaluation of a neck deep vein thrombosis revealed pulmonary nodules, prompting her primary care physician to refer her to the ED.

**Figure 1:**
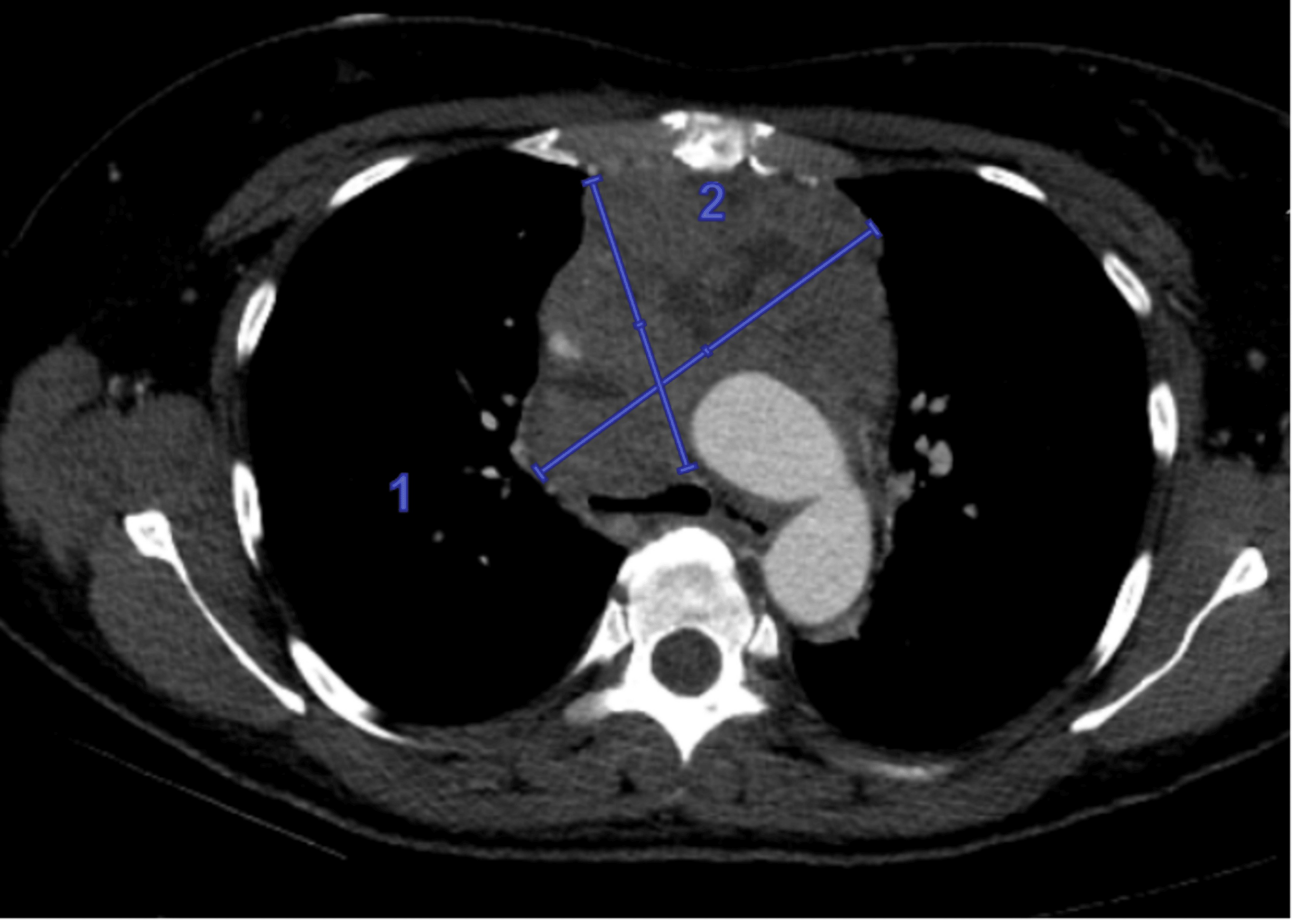
Large superior mediastinal mass seen on contrast-enhanced axial computed tomography (blue lines numbered 1 and 2, approximately 10 × 7.3 × 12.3 cm) with the extensive involvement of vascular structures (the superior vena cava, brachiocephalic vein, and ascending aorta) and adjacent airways (the trachea and right main bronchus), consistent with malignancy.

Imaging identified a new anterior mediastinal mass as shown in Figure 1, with associated lymphadenopathy. Hematology/oncology and the multidisciplinary thoracic team were consulted. Biopsies of lymph nodes and bone marrow revealed a poorly differentiated epithelial malignancy. Due to concern for evolving superior vena cava syndrome, interventional radiology performed catheter-directed thrombolysis of the left innominate vein. The patient was monitored in the intensive care unit with the thrombolysis catheter left in situ. However, tissue plasminogen activator was discontinued due to bleeding complications. Stenting was deferred in favor of initiating chemotherapy, which was believed to better address the underlying malignancy and SVC obstruction.

The patient was transferred to the progressive care unit, where she received her first dose of chemotherapy. During her hospitalization, she developed volume overload, which was managed with intravenous furosemide. CT imaging revealed bilateral pulmonary infiltrates, and she developed sinus tachycardia, attributed to systemic inflammation from malignancy. She received supportive care, including Granix (tbo-filgrastim) and blood and platelet transfusions per oncology recommendations, and anticoagulation was held due to thrombocytopenia. The patient showed clinical improvement and was discharged in stable condition with close outpatient follow-up.

**Figure 2:**
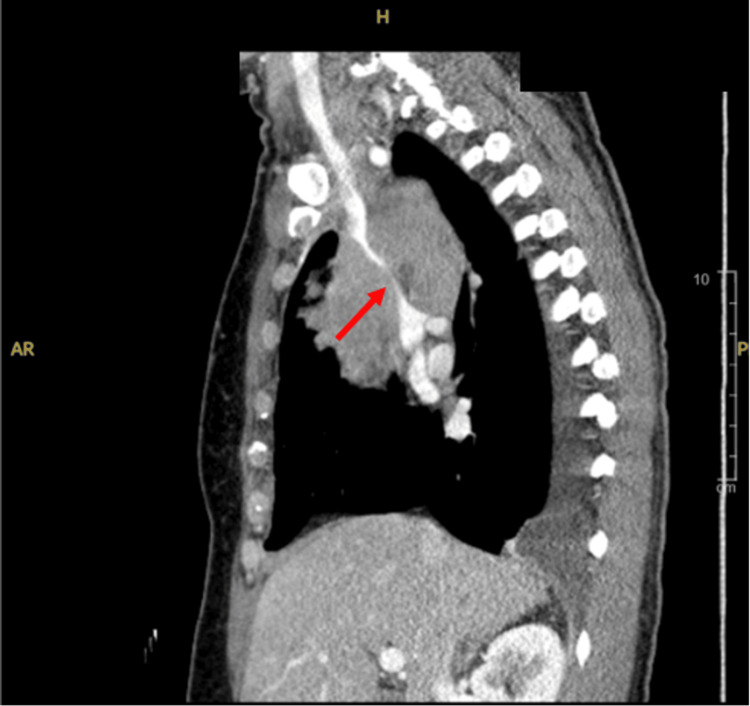
The sagittal computed tomography findings are consistent with superior vena cava (SVC) syndrome, characterized by the near-complete compression of the SVC by a large, invasive anterior mediastinal mass. The SVC appears severely narrowed to a slit-like configuration, and there is an encasement of the left brachiocephalic vein, likely resulting in occlusion.

Two weeks later, she returned to the ED with neck tightness, shortness of breath, and new-onset hallucinations and confusion following chemotherapy. CT angiography showed an occlusive thrombus in the left internal jugular vein and a 7.9 cm anterior mediastinal mass causing significant SVC compression, as shown in Figure 2. Biopsy confirmed a nonseminomatous germ cell tumor (NSGCT). She was initiated on platinum-based chemotherapy (bleomycin, etoposide, and cisplatin {BEP} regimen) via a peripherally inserted central catheter, with improvement in symptoms and radiographic response. She also received antibiotics for multifocal pneumonia.

**Figure 3:**
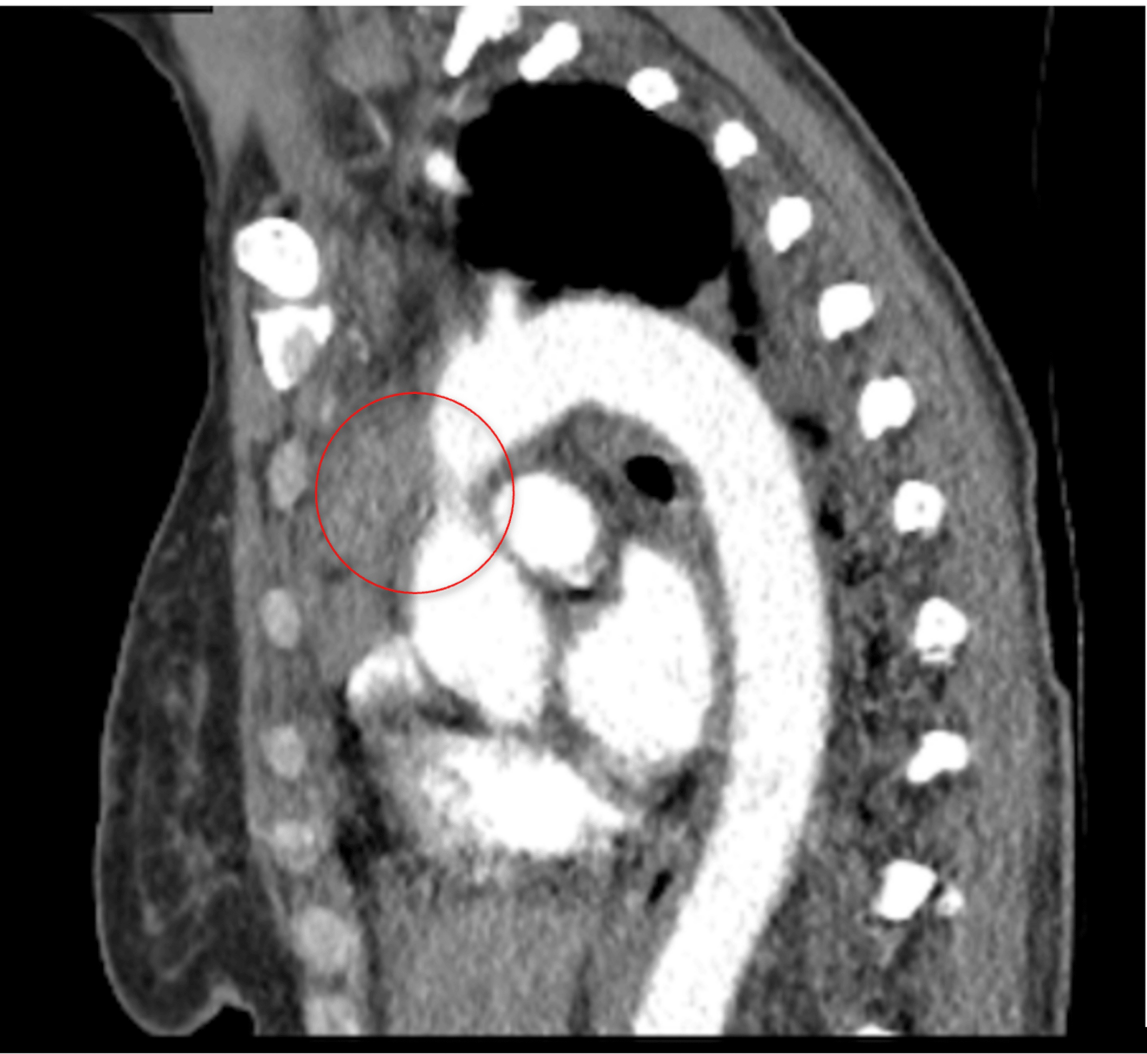
Sagittal computed tomography angiogram showing further progression of the infiltrative mediastinal mass, now extending into the pericardium and encasing the aortic arch as circled in red.

After six months of chemotherapy, the patient again presented to the ED with acute hypoxia and fever during outpatient follow-up. Imaging demonstrated bilateral pneumonia, small pleural effusions, the progression of the mediastinal mass, and new hepatic metastases. She improved with intravenous antibiotics and supplemental oxygen and was discharged on home oxygen therapy. However, three days later, she returned to the ED with worsening shortness of breath, neck and chest congestion, and profound fatigue. Laboratory values upon re-admission are listed in Table 1 with reference ranges. CT angiogram showed further progression of the infiltrative mediastinal mass, now extending into the pericardium, encasing the aortic arch, and worsening SVC stenosis as shown in Figure 3.

**Table 1:** Patient’s laboratory values upon re-admission.

Laboratory test	Result	Reference range
Hemoglobin	7.0 g/dL	12.0-16.0 g/dL
Platelet count	47 × 10³/µL	150-450 × 10³/µL
White blood cell count	2.0 × 10⁹/L	4.0-10.0 × 10⁹/L
Blood urea nitrogen	37 mg/dL	7-20 mg/dL
Creatinine	1.2 mg/dL	0.6-1.3 mg/dL
Procalcitonin	0.5 ng/mL	<0.1 ng/mL
Lactate	2.6 mmol/L	0.5-2.2 mmol/L

She was admitted for supportive management and treatment of acute-on-chronic anemia, receiving one unit of A+ packed red blood cells. Hematology/oncology initiated intravenous corticosteroids and oral imatinib. Despite these efforts, her respiratory status continued to deteriorate. Pulmonology recommended continued oncologic management. Interventional radiology placed an SVC stent to relieve vascular obstruction; however, her condition worsened, requiring high-flow nasal cannula oxygen therapy. Given the progression of the disease and poor prognosis, palliative care was consulted. After extensive goals-of-care discussions, the patient elected for comfort-focused management and was discharged to an inpatient hospice facility.

## Discussion

This case underscores the aggressive clinical course and multisystem involvement characteristic of nonseminomatous primary mediastinal germ cell tumors, particularly in the rare setting of a female patient. These tumors are not only biologically aggressive but also strategically located in a confined anatomic compartment, allowing even moderate tumor growth to result in life-threatening complications. Delayed diagnosis is common due to vague and nonspecific initial symptoms such as dyspnea, cough, or upper extremity edema, which are often misattributed to more common cardiopulmonary conditions. In this case, the initial respiratory symptoms and facial swelling reflected early signs of superior vena cava syndrome, which progressed due to unchecked tumor growth and venous obstruction.

SVC syndrome is a hallmark complication of mediastinal masses, occurring when the tumor compresses or invades the SVC, leading to increased venous pressure in the upper body. This results in facial and neck swelling, dilated thoracic and cervical collateral veins, dyspnea, orthopnea, and, in severe cases, cerebral edema or airway compromise. The simultaneous development of internal jugular vein thrombosis further compounded venous obstruction and highlights the tumor’s propensity for both compressive and thrombotic vascular complications. These findings reflect not just mechanical obstruction but also a prothrombotic paraneoplastic state.

Systemic complications further illustrate the tumor’s widespread impact. Chemotherapy-induced immunosuppression predisposed the patient to multifocal pneumonia and severe neutropenia, while profound anemia likely reflected both marrow suppression and tumor-associated inflammation [6]. These hematologic and infectious complications are common during platinum-based chemotherapy, but they significantly contribute to morbidity and may delay further oncologic treatment. Platinum-based chemotherapy uses platinum-containing compounds, such as cisplatin, carboplatin, and oxaliplatin, to damage and kill cancer cells. These drugs work by forming platinum-DNA adducts that block DNA replication and transcription, ultimately leading to cell death [7]. Neurologic and cardiovascular complications added further complexity. Chemotherapy-induced cognitive impairment has been reported, which has led to decreased memory, executive function, and inattention. Chemotherapy-associated neurotoxicity, manifesting as confusion and hallucinations, though transient, may reflect high-dose cisplatin effects or metabolic derangements in a critically ill host [8]. The development of sinus tachycardia, responsive to beta-blockade, likely reflects a combination of tumor burden, anemia, hypoxia, and autonomic stress, frequent but often underappreciated sequelae in this setting.

Despite initial radiologic response to chemotherapy, the patient experienced continued disease progression, including hepatic metastases and pericardial involvement, features indicative of a poor prognosis. Nonseminomatous PMGCTs are known for their chemoresistance, early dissemination, and limited surgical resectability due to their location and vascular invasion. In the mediastinum, these germ cell tumors are associated with poorer outcomes than their gonadal counterparts, with significantly lower complete response rates and survival. In one retrospective cohort of poor-risk NSGCTs, mediastinal tumors exhibited a complete response rate of just 38%, compared to 61% for retroperitoneal and 38% for testicular primaries. Patients with mediastinal NSGCTs experienced the lowest event-free survival of all locations and were more prone to relapse following chemotherapy [9].

This case not only exemplifies the diverse complications associated with PMGCTs but also serves as a reminder of the importance of early recognition, multidisciplinary care, and the anticipatory management of complications such as SVC syndrome, immunosuppression, and treatment-limiting toxicities. For rare presentations in women, a high index of suspicion is essential to avoid delays that can drastically affect outcomes.

## Conclusions

In this rare case, we observe the progression of a primary mediastinal nonseminomatous germ cell tumor in a female patient, a clinical scenario that is exceptionally uncommon. These tumors are multifactorial due to their local invasiveness, vascular compression, and rapid progression. Early imaging, prompt tissue diagnosis, and coordinated multidisciplinary management are essential for optimizing outcomes. This case highlights the need for heightened clinical suspicion in atypical populations, the importance of recognizing SVC syndrome, and the challenges of managing chemotherapy-related complications while anticipating multisystem involvement during treatment.
